# Specific Combination of Salvianolic Acids As Core Active Ingredients of Danhong Injection for Treatment of Arterial Thrombosis and Its Derived Dry Gangrene

**DOI:** 10.3389/fphar.2017.00361

**Published:** 2017-06-13

**Authors:** Tiechan Zhao, Lianying Chang, Boyong Zhang, Ming Lu, Xiaoyi Wang, John O. Orgah, Yuefei Wang, Xiaoxuan Tian, Jing Yang, Guanwei Fan, Boli Zhang, Yan Zhu

**Affiliations:** ^1^Tianjin State Key Laboratory of Modern Chinese Medicine, Tianjin University of Traditional Chinese MedicineTianjin, China; ^2^Research and Development Center of TCM, Tianjin International Joint Academy of Biotechnology and MedicineTianjin, China; ^3^Tufts Medical Center, Molecular Cardiology Research Institute, Tufts University School of MedicineBoston, MA, United States

**Keywords:** anti-platelet therapy, G-protein-coupled receptors, gangrene, danhong injection, salvianolic acid

## Abstract

Although single-targeting anti-platelet agents are used extensively in clinics, their limitations in resistance and bleeding have started a trend of combination therapy. Danhong injection (DHI) is a widely prescribed injection medicine for cardiovascular and cerebrovascular diseases in China. However, its precise clinical efficacy and functional components remain unexplored. In this study, we investigated the anti-thrombotic role and its chemical basis of DHI. In a photochemically-induced thrombosis model, DHI effectively dissolved thrombus and ameliorated its derived dry gangrene. DHI inhibited multiple GPCR agonists-induced platelet adhesion, aggregation and downstream Ca^2+^ and cAMP signaling pathways. A functional screen of DHI library identified its major active components as a cluster of seven salvianolic acids. A combination of salvianolic acid A and C synergistically inhibited platelet aggregation *in vitro* while salvianolic acid B antagonized this effect. Our study revealed the anti-thrombotic activity of DHI. The multi-targeting mechanism of DHI proves the effectiveness of a natural anti-thrombotic combination therapy. The identification of salvianolic acids as a core anti-thrombotic activity of DHI and the discovery that their different combinations could either synergistically or antagonistically provide a better guidance for safer clinical application and paves the way for further development of DHI.

## Introduction

Platelets play a pivotal role in arterial thrombosis and inflammation after an atherosclerotic plaque rupture, either spontaneously or in the setting of percutaneous coronary intervention (PCI) (Yousuf and Bhatt, 2011). Platelet adhesion and aggregation at sites of vessel-wall injury are critical for the arrest of bleeding and for the development of vaso-occlusive thrombi at sites of atherosclerotic-plaque rupture.

Arterial thrombosis is a major cause of arterial embolism, potentially causing infarction of almost any organ in the body. If the blood flow is interrupted other than severe bacterial infection, it may cause dry gangrene (Gardner and Afaq, 2008). Dry gangrene begins at the distal part of the limb due to ischemia, and often occurs with the incidence of vascular disease, e.g., arteriosclerosis, thrombosis, or destruction of tissue by injury (Korzon-Burakowska and Dziemidok, 2011). Dry gangrene is mainly caused by arterial occlusion.

G protein-coupled receptors (GPCRs) on the membrane of platelet, such as adenosine diphosphate (ADP) receptor, thrombin receptor and thromboxane A_2_ receptor, are key players in the thrombotic process (Dowal and Flaumenhaft, 2010; Piazza and Goldhaber, 2010). Current used antiplatelet drugs targeting GPCRs, such as the cyclooxygenase inhibitor aspirin and the P2Y12 inhibitor clopidogrel, have led to considerable improvements in the management of thrombotic disease. However, these anti-platelet agents also have their limitations. For example, treatment with aspirin may develop aspirin resistance or may increase the risk of bleeding (Lim, 2011).

Because platelet activation is determined by multiple receptor-mediated signaling pathways, clinical studies have evaluated the efficacy of multidrug administration in the prevention of atherothrombotic complications. Ca^2+^-pump inhibitors have been widely used in the study of the role of intracellular Ca^2+^ in various cellular events, including platelet activation, aggregation and secretion. Production of cAMP had the opposite effects on ADP induce platelet activation.

Although single-targeting anti-platelet agents are used extensively in clinics, their limitations have started a trend of combination therapy. For example, bleeding risk of warfarin (Liu H. T. et al., 2013), resistance of aspirin (Liu X. et al., 2013), and clopidogrel resistance (Michelson, 2010). Chinese herbal medicine has been a rich source of drug candidates that is considered as a natural multiplex medicine. Most Chinese medicine injections (CMIs) exert therapeutic effects based on the synergic effects of their multi-components and multi-targets. Danhong injection (DHI), made from aqueous extracts of *Salvia miltiorrhiza* and *Carthamus tinctorius*, is one of the most prescribed CMIs in China. In addition to its wide hospital use in China, the efficacies of DHI for traumatic intracranial hematoma (Liu X. et al., 2013), acute cerebral infarction (Vayvada et al., 2013) and cerebral damage during on-pump coronary bypass graft surgery (Liu H. T. et al., 2013) have been demonstrated in clinical trials. Through clinical observation, DHI have a certain effect on platelet activation in patients of acute coronary syndrome (Chen et al., 2009; Sun et al., 2009). However, its systemic and interrelated actions in thrombosis model have not yet been clarified (Chen et al., 2011). Previously, we have identified 11 polyphenolic acids in DHI via ultra-performance liquid chromatography (UPLC) coupled with ultraviolet (UV) detection (Liu H. T. et al., 2013). With a newly developed proton H-1detection nuclear magnetic resonance (1H NMR) profiling method, we simultaneously identified and quantified 23 primary metabolites and 7 polyphenolic acids in DHI (Jiang et al., 2014). Recently published papers showed that a total of 63 compounds (33 phenolic acids, 2 C-glycosyl quinochalcones, 6 flavonoid O-glycosides, 4 iridoid glycosides, 6 organic acids, 5 amino acids, and 3 nucleosides) in DHI were characterized and identified (Zhang et al., 2016).

Salvianolic acids (SAs) are a series of the most abundant ingredients in *Salvia miltiorrhiza*, their reported pharmacological effects include potent ischemia/reperfusion protection (Xue et al., 2014), anti-platelet aggregation (Lee et al., 2014; Yu et al., 2014), anti-thrombosis (Huang et al., 2010), and neuroprotection (Du and Zhang, 1997; Brouns and De Deyn, 2009). To date, over 20 SA compounds have been identified by phytochemical isolation or chromatographic separation, including tanshinol (TSL), protocatechuic aldehyde (PCA), rosmarinic acid (RA), caffeic acid (CA), lithospermic acid (LA), salvianolic acid A (SAA), salvianolic acid B (SAB), salvianolic acid C (SAC), and salvianolic acid D (SAD) (Hu et al., 2005; Liu et al., 2007). It was reported that SAA exert anti-thrombotic activity by inhibiting phosphatidylinositol 3-kinase (PI3K) pathway of platelet (Huang et al., 2010). SAB significantly decreases the apoptosis of myocardial cells after ischemia-reperfusion by increasing the level of antioxidant substances. However, their precise roles and molecular targets in thrombosis disease have not yet been clarified.

This present study aimed at defining the efficacy and chemical basis of DHI in thrombotic disease. We showed that DHI could dissolve photochemically-induced artery thrombus *in vivo* and prevent dry gangrene formation in a rat model. We further demonstrated that DHI acted through inhibition of activated multiple platelet GPCRs and identified its anti-platelet active fractions as mixtures of SAs. Moreover, using purified SAs, we defined a specific combination of SAA and SAC as the most potent anti-platelet aggregation agents.

## Materials and methods

### Experimental animal

Sprague-Dawley rats (SD, both sexes, body mass 200–220 g) were purchased from Beijing HFK Bioscience Co., Ltd (License Numbers SCXK-(jun) 2007-004) and maintained under specific pathogen-free conditions at Tianjin International Joint Academy of Biomedicine (TJAB). They were housed in groups in cages within the facility and maintained on normal rat chew and purified water. Blood sample was collected from either male or female rats for platelet isolation and preparation for the *in vitro* assay of platelet adhesion, platelet aggregation, measurement of cAMP and determination of [Ca^2+^]_i_. Male rats were used for *in vivo* experiment (thrombosis animal model). All animal experiments were performed in accordance with the international regulations and approved by the animal care and use committee of TJAB (#TJU20160021).

### Reagents

ADP (catalog No.A2754), thrombin (catalog No.T1063), heparin (catalog No.H3149), U46619 (catalog No.D8174), Aspirin (catalog No.A5376), ticagrelor (catalog No. CDS023238), EGTA (catalog No.03777), EDTA (catalog No. EDS), rose Bengal (4, 5, 6, 7-tetrachloro-2′, 4′, 5′, 7′-tetraiodofluorescein, catalog No.198250) and Prostaglandin E1 (catalog No.P5515) were purchased from Sigma-Aldrich (St. Louis, USA). DHI (Batch number: 12071086) was supplied by Heze Buchang Pharmaceutical Co. Ltd. (Shandong, China). The original manufacture ampoule of DHI was the stock solution which was diluted with corresponding buffers used in each test. Direct cAMP ELISA kit (catalog No.ADI-900-066) was purchased from Enzo Life Science (San Diego, USA). Fluo-3/AM (catalog No. S1056) was purchased from Beyotime Co. (Shanghai, China). Isoflurane (catalog No. 207150301) was purchased from RWD Life Science Co. (Shenzhen, China).

### Induction of right external iliac artery thrombosis

Right external iliac artery thrombosis was induced by laser-photochemical injury. Rats were anesthetized with 3% isoflurane in 69% N_2_O/30% O_2_ at continuous inhalation using isoflurane-vaporizer (Matrx VIP 3000, RWD Life Science Co. Shenzhen, China). The right external iliac artery was dissected free of underlining tissues and arterial blood flow was measured with a laser Doppler scanner and digital recording system (laser Doppler line Scanner/Perfusion Imager, Moor Instruments Inc. Delaware, USA). To induce endothelial injury, the right external iliac artery was trans-illuminated continuously with a 1.5-mV, 540-nm green laser (Melles Griot, Carlsbad, CA) from a distance of 6 cm, and photosensitive dye rose Bengal (90 mg/kg) was injected via tail vein. Blood flow was monitored continuously for 90 min, at which time the experiment was terminated. Photochemically-induced rats arterial thrombus were then divided into 5 groups: model (saline), DHI low (1.75 mL/kg), DHI medium (5.25 mL/kg), DHI high (15.75 mL/kg), and heparin (2,500 U/kg). Rats that were surgically operated but without photochemically-induction served as normal control. Rats which in DHI groups were treated with DHI via i.v. once a day for 7 days. After 7 days, all animals were examined for blood clot formation and the degree of dry gangrene as described below.

### Measurement of blood clots and dry gangrene

The SD rats were anesthetized with 3% isoflurane in 69% N_2_O/30% O_2_ at continuous inhalation. Right external iliac artery was dissected free of underlining tissues and a small section of artery was cut down at the laser irradiated point where arterial clot formed. Quantitative measurements of arterial clot area were done by imaging through an inverted microscope (Nikon Instech Co. Ltd. Japan), and subsequently calculated using the software inbuilt by Nikon (see Supplementary Material for details).

Dry gangrene was classified according to clinical diagnosis guidelines with minor modifications in an order of decreased severity: Gangrene > Tissue ulcer > Cellulitis/Tissue abscess > Tissue (dry and shrunken) (see Supplementary Material for details).

### Platelet preparation

After SD rats were anesthetized with 3% isoflurane in 69% N_2_O/30% O_2_ at continuous inhalation, blood was collected from the abdominal aorta to a tube containing anti-coagulant with 10% Acid-citrate-dextrose buffer (ACD, 38 mM citric acid, 75 mM sodium citrate, and 124 mM dextrose). It was then centrifuged at 1,270 r/min for 12 min at room temperature. Platelet-rich plasma was acidified to pH 6.5 with ACD and platelets were pelleted through plasma by centrifugation at 2,560 r/min for 10 min and re-suspended in buffer A (130 mM NaCl, 10 mM sodium citrate, 9 mM NaHCO_3_, 6 mM dextrose, 0.9 mM MgCl_2_, 0.81 mM KH_2_PO_4_, 10 mM Tris, pH 7.35 ~ 7.45) (Salzman et al., 1969).

### Platelet adhesion assay

Plates were coated with 50 μL/well of human fibrinogen (20 mg/ml, overnight at 4°C). Before use, the wells were washed twice with 0.9% NaCl and non-specific adhesion was blocked by incubation with 0.35% BSA (1 h at 37°C). A 50 μl platelet suspension (2 × 10^7^ platelets/mL), 1 mM Ca^2+^ 0.35%, BSA and different concentration of DHI were added to each well and incubated for 20 min at 37°C. Then, 10 μM of ADP or thrombin (0.1 U/mL) were added to each well and incubated (30 min, 37°C). The plate was washed twice with Buffer A (120 μL/well) and then platelet number was determined at a detection wavelength at 405 nm by a plate reader (FlexStation® 3, Molecular Devices).

### Platelet aggregation assay

Washed platelets were prepared and re-suspended at concentration of 1 × 10^8^ platelets/mL in buffer A and 1.8 mM CaCl_2_ was added just prior to assay. After 100 μL (~1 × 10^7^ platelets) platelets were dispensed into 96-well microplate, different concentration of DHI or buffer A was added to bring the final volume to 150 uL and incubated with shaking at 37°C for 10 min in FlexStation® 3 plate reader. ADP (25 μM), thrombin (0.5 U/mL) or U46619 (11.4 μM) were dissolved in buffer A, and added to bring the final volume to 200 uL. Plate was incubated at 37°C with shaking in a kinetic mode in FlexStation® 3 Response was real-time measured for 30 min with a detection wavelength at 405 nm. The lowest point set as the maximum aggregation.

$$\begin{array}{l} \text{MPAR}(\text{maximum platelet aggregation rate})\\ \;=\frac{\text{ODmaximumaggregation}-\text{ODbeginning}}{\text{ODmaximumaggregation}}\;\times \text{ 100}\% \end{array}$$

### Measurement of cAMP

Washed platelets (1 × 10^8^ platelets/mL, without PGE_1_ in the preparation) were pre-incubated with different concentrations of DHI solution with buffer A (final volume 150 μL) in the presence of 2 mM CaCl_2_ with shake (37°C and 100 r/min) for 10 min, then 20 μM ADP was added for 8 min. Equal volume of 0.1M HCl was added with 5 times repeated freezing and thawing, and centrifuged at 3,630 r/min for 10 min, at room temperature. The cAMP concentration was measured according to the manufacture's instruction [see Supplementary Material for details. Direct cAMP ELISA kit, Enzo Life Science (San Diego, USA)]. Forskolin (10 μM) was used as a positive control for a stimulated production of cAMP.

### Determination of intracellular Ca^2+^ concentration

The intracellular Ca^2+^ concentration ([Ca^2+^]^i^) was determined with Fluo-3/AM as described previously (Puri et al., 1989). Platelets were centrifuged at 1,260 r/min for 8 min and re-suspended at concentration of 1 × 10^8^ platelets/ml in modified Tyrodes buffer (150 mM NaCl, 3 mM KCl, 1 mM MgCl_2_, 10 mM Hepes, 0.5 mM dextrose, 0.1% BSA, PH 7.4) supplemented with 10 U/mL heparin, 0.2 U/mL Apyrase, and 1 μM PGI_2_, incubated for 8 min in room temperature, then washed (Bednar et al., 1995). Platelets were re-suspended in modified Tyrodes buffer containing 2.5 mM probenecid, 0.02% pluronic and 10 μM Fluo-3/AM, 0.5 μM PGI_2_, 0.02 U/ml apyrase and shaked (30min, 100 r/min) at 37°C. Different concentrations of DHI was added to platelets and incubated for 15 min. After washing out unbound dye with 0.5 μM PGI_2_, platelets were re-suspended in Tyrodes buffer and dispensed into 96-well microplate. [Ca^2+^]_i_ was measured by Fluo-3/AM fluorescence (EX 488 nm, EM 525 nm) in flex mode of FlexStation® 3, with a sampling rate of one per sec. with 2 mM CaCl_2_ and 15 μM ADP added automatically after 10 min incubation.

### UPLC analysis conditions

The chromatographic analysis of DHI fractions was performed on an ultra-performance liquid chromatography system (Waters Corp., Milford, MA, USA) equipped with a binary gradient solvent pump, an automatic sampler, a column oven and a diode array detector. Chromatographic separation was carried on an Acquity UPLC HSS T3 column (2.1 × 100 mm, 1.8 μm) at 40°C. The mobile phase was composed of 0.1% formic acid aqueous solution (A) and acetonitrile (B), and the gradient program was employed as following: 0~7 min, 3~19% B; 7 ~ 13 min, 19% B; 13 ~ 18 min, 18 ~ 25% B; 18 ~ 25 min, 25 ~ 90% B. The flow rate of the mobile phase was set at 0.4 mL/min and the injection volume was 2 μL. The detection wavelengths were 254 and 286 nm.

### Mass spectrum analysis

The Mass Spectrum (MS) analysis of the potentially active fractions of DHI was performed as described previously. Analysis was performed on a Waters Xevo G2-S high definition mass spectrometer system (Waters, Milford, MA, USA) equipped with an electrospray ion (ESI) source operating in negative ion modes. The capillary voltage was set at –2.0 kV. The sampling cone voltage was set at 30 V. The flow of nitrogen desolation gas was set at 800 L/h with a temperature of 400°C. The flow of nitrogen cone gas was set at 50 L/h and the source temperature was set at 100°C. The mass scan range was from 50 to 1,500 Da in MS^E^ acquisition mode. For acquisition of the accurate mass-to-charge ratio, the MS was corrected during data acquisition using a lock mass of leucine-enkephalin (LE) at a concentration of 1 ng/mL via a Lock Spray™ interface at a flow rate of 10 μL/min, with monitoring for reference ions in the negative ion mode ([M – H]– = 554.2615) to ensure accuracy during MS analysis. All the data were acquired and processed by Masslynx V4.1 software.

### Preparation of DHI chemical library

The established Prep-HPLC method was performed as described previously (Wang et al., 2016). The preparation of DHI fractions was performed on a preparative high performance liquid chromatography (Prep-HPLC) system (Waters Corp., Milford, MA, USA) equipped with a binary gradient solvent pump, an ultraviolet detector, a manual injector and an automatic fraction collection device (EYELA Fraction Collector DC-1500). Chromatographic separation was carried out on a Zorbax SB-C18 column (21.2 × 250 mm, 7 μm) at room temperature. The mobile phase was composed of methanol (A) and 0.1% formic acid aqueous solution (B), and the following gradient program was employed: 0–6 min, 5% A; 6–81 min, 5–70% A; 81–87 min, 70–95% A. The first fraction was collected by 0–6 min, the second to twenty-sixth fractions were collected by 6-81 min every 3 min, and the last fraction were collected by 81–87 min, numbered as Fraction no. 1–27. Appropriate freeze-dried powders of the 27 DHI fractions were accurately weighed and diluted with methanol to give a concentration of 10 mg/mL stock solutions. For UPLC analysis, the stock solution of each fraction was diluted with 30% methanol to prepare 5 mg/mL sample solutions. For platelets aggregation assay, the stock solution of each active fraction was diluted with buffer A to prepare a 100 μg/mL sample solution. For MS analysis, the stock solution of each active fraction was diluted with 50% methanol to prepare a 0.5 mg/mL sample solution.

### Synergy determination

The isobologram analysis for the combination study was based on a method by Chou-Talalay to determine combination indices (CI). The data obtained with the platelet aggregation assay was plotted as aggregation rate vs. concentration of drugs. The data was then converted to Fraction affected (Fa; range 0–1; where Fa = 0 represents 100% viability and Fa = 1 represents 0% viability) and analyzed with the CompuSyn™ program (Biosoft, Ferguson, MO) based upon the Chou and Talalay median effect principle (Chou and Talalay, 1984). The combination index (CI) values reflect the ways of interaction between two drugs, which take into account both potency (inhibitory concentration values) and shape (slope, m) of dose-effect curve. CI <1 indicates synergism, CI = 1 indicates an additive effect, and CI > 1 indicates antagonism.

### Statistical analysis

Data are reported as mean ± standard errors. Statistical differences were assessed using one-way ANOVA followed by *post-hoc* analysis with Dunnett's *t*–test. *P* < 0.05 was considered statistically significant.

## Results

### DHI dissolved photochemically-induced arterial thrombus in a rat model

**Figure 1:**
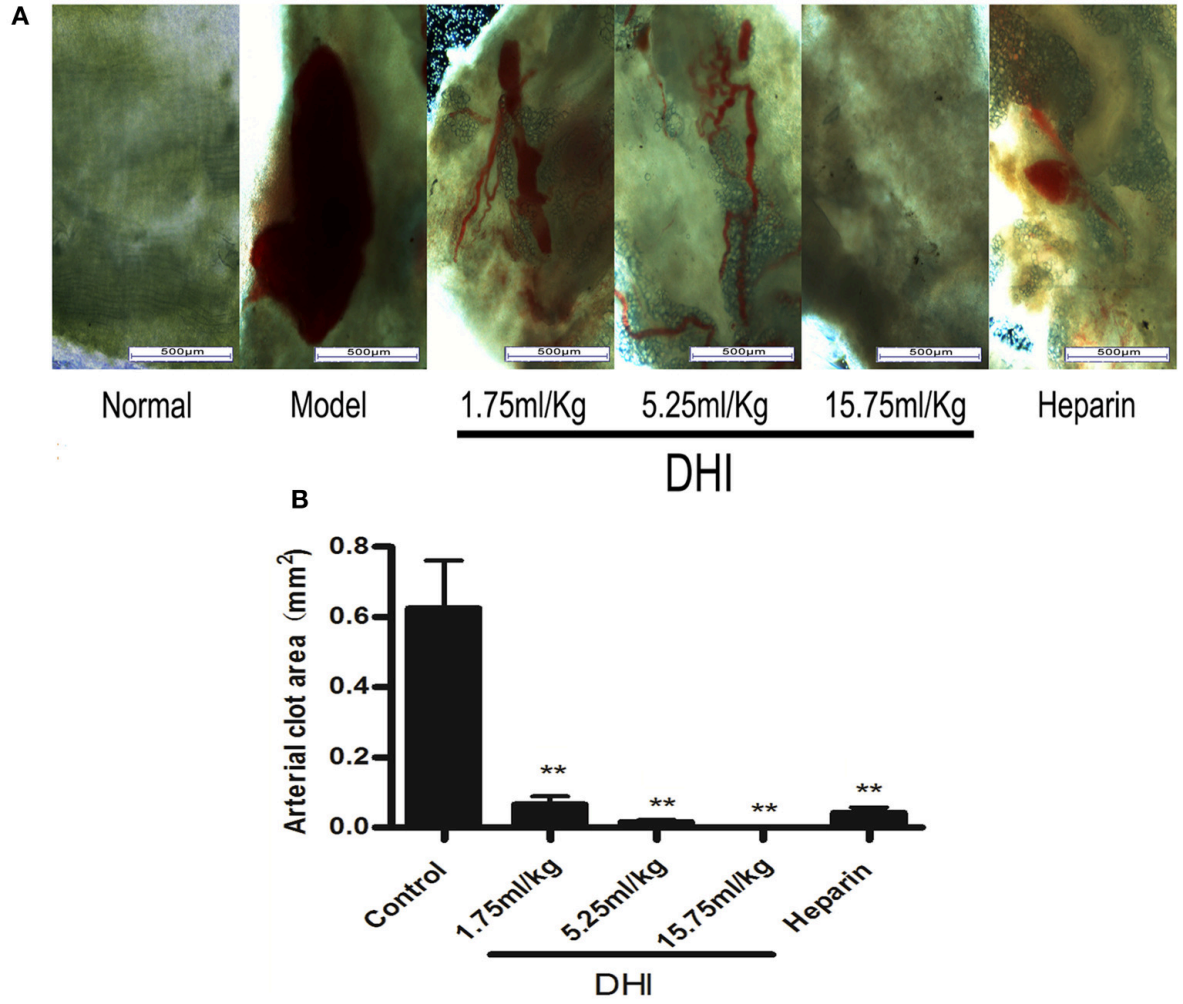
DHI dissolved laser-induced arterial thrombus in a rat model. **(A)** Photomicrographs of arteria thrombosis. Laser activation of rose Bengal dye caused a clearly visible blood clot in the rat iliac arteries. Three doses of DHI (1.75, 5.25, and 15.75 mL/kg) dissolved the clots. Heparin (2,500 U/kg) was used as a positive control. DHI or heparin was injected for 7 days as follow-up treatment. **(B)** Bar graph quantifying the arterial clot area. (Data are expressed as mean ± S.D. of 8 rats per group, ^**^P < 0.01).

First of all, in order to prove the efficacy of DHI on thrombotic diseases, we adopted a photochemically-induced arterial thrombus formation model (Dayal et al., 2006). Right external iliac artery of rats was trans-illuminated continuously with a 1.5-mV, 540-nm green laser from a distance of 6 cm for 90 min and rose Bengal (90 mg/kg) was injected into tail vein to induce arterial clot (0.624 ± 0.136 mm^2^). As a positive control, 2,500 U/kg heparin decreased the arterial clot area to 4.18 × 10^−3^ mm^2^ (*n* = 8, *p* < 0.01) (Figure 1). In comparison, DHI (1.75 mL/kg-15.75 mL/kg) dose-dependently reduced the arterial clot area from 0.624 ± 0.136 mm^2^, to 6.58 ± 2.3 × 10^−3^ mm^2^, respectively (*n* = 8, *p* < 0. 01).

### DHI ameliorated symptoms of dry gangrene caused by arterial thrombus

**Figure 2:**
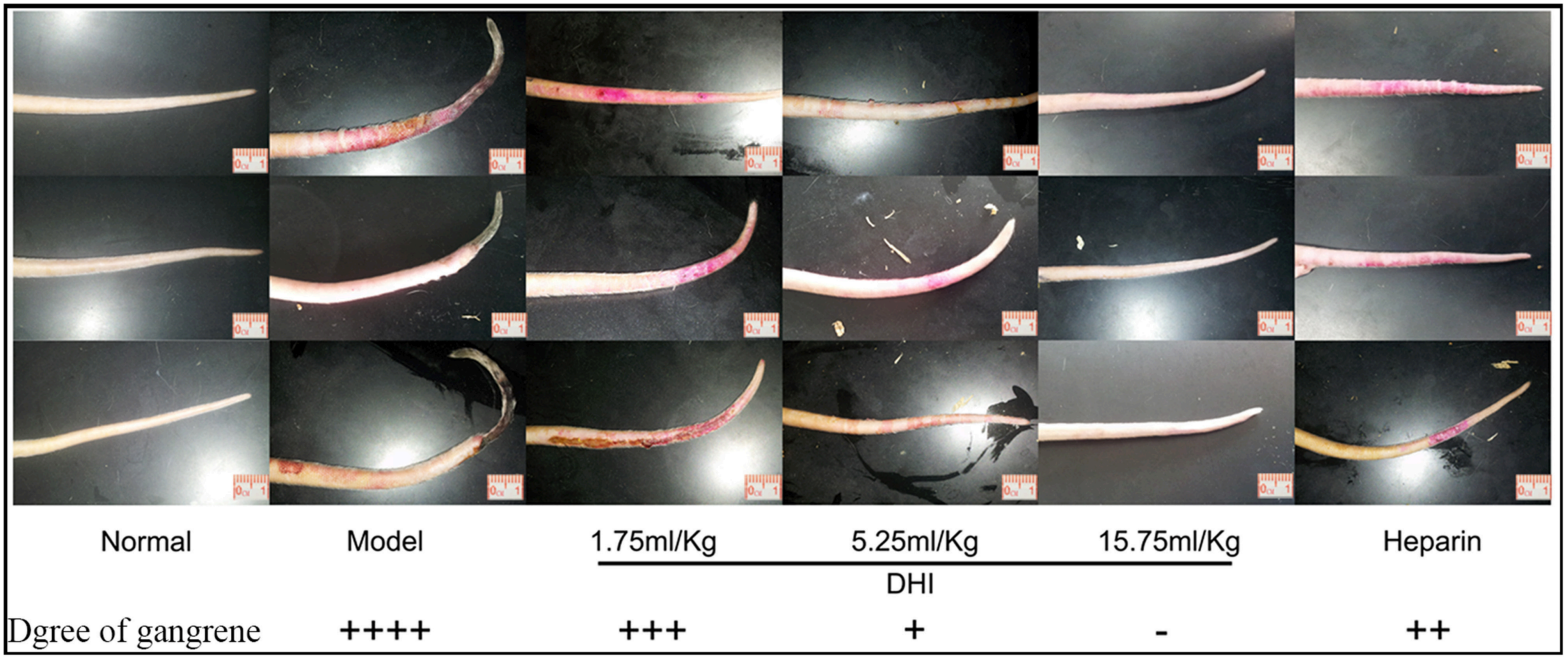
DHI ameliorates symptoms of dry gangrene caused by artery occlusion. There was obvious necrosis of the tail in rats that without treatment. The three examples were individual animals by the same group setting. Symptom of dry gangrene was reduced by treatment with different dose of DHI. Low dose DHI (1.75 mL/kg) relieved the tissue ulcer. Medium dose of DHI (5.25 mL/kg) reduced the lesions to dry and shrunken disposition. The symptom of dry gangrene was resolved by high dose of DHI (15.75 mL/kg). As a positive control, heparin (2,500 U/kg) also reduced the lesions to dry and shrunken disposition similar to medium dose of DHI (8 rats per group).

Blood clots in iliac artery obstructed free flow of blood to the tail leading to dry gangrene. The symptoms of dry gangrene (or tissue ulcer) were reduced significantly to mild tissue ulcer after treatment with low concentration DHI (1.75 mL/kg, *n* = 8). Medium concentration of DHI (5.25 mL/kg, *n* = 8) reduced the lesions to dry and shrunken constitution. The dry gangrene was completely healed by high concentration of DHI (15.75 mL/kg, *n* = 8). In comparison, the positive control (heparin) reduced the gangrene to dry and shrunken as similar as the medium dose of DHI (Figure 2).

### DHI inhibited platelet adhesion *in vitro*

**Figure 3:**
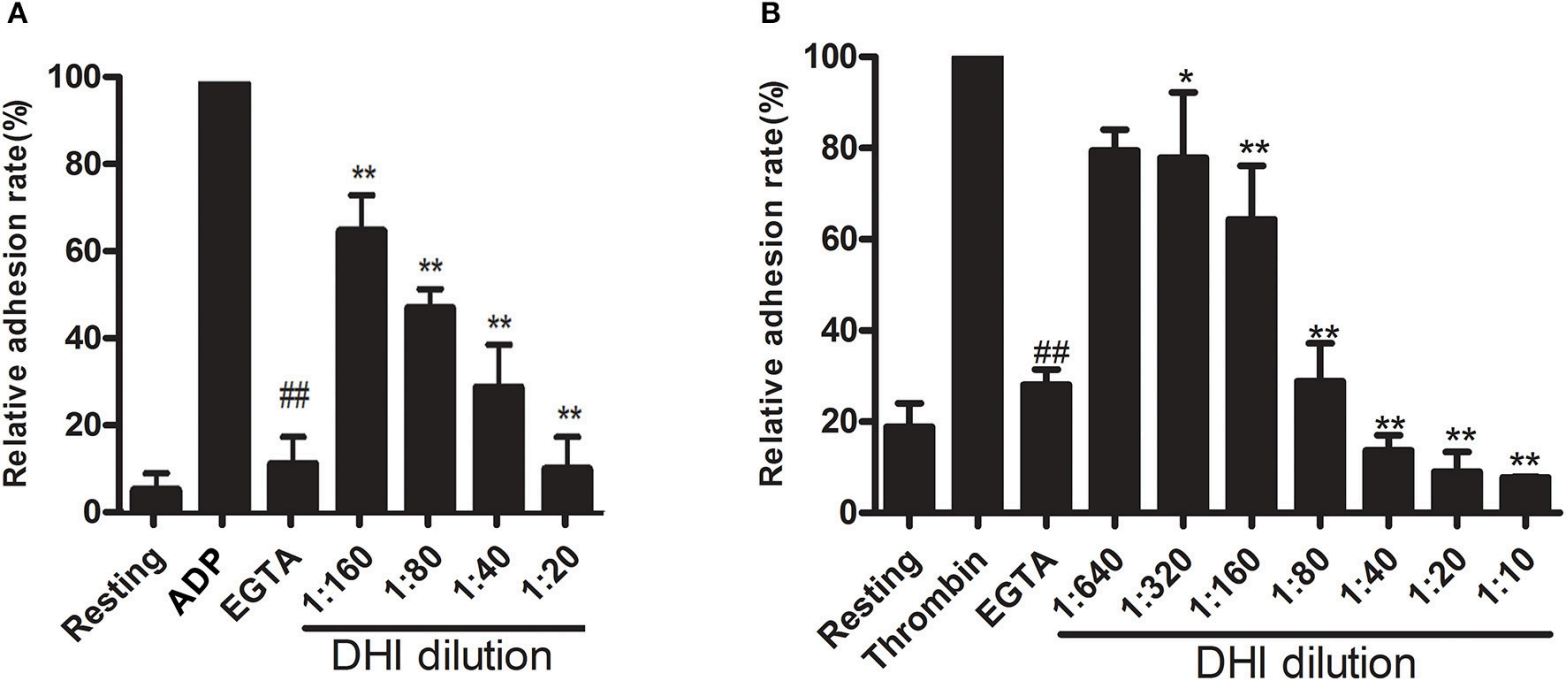
DHI inhibited platelet adhesion. **(A)** Platelets (1 × 10^8^ platelets/mL) were pre-incubated for 10 min with a series of DHI concentration (1:20–1:160) and allowed to adhere to fibrinogen-coated wells containing ADP (10 μM) for 15 min. **(B)** Platelets (1 × 10^8^ platelets/mL) were pre-incubated for 10 min with a series of DHI concentration (1:10–1:640) and allowed to adhere to fibrinogen-coated wells containing thrombin (0.1 U/mL) for 15 min. Platelet adhesion was shown as a percentage of the maximal signal induced by ADP or thrombin in control conditions. EGTA was used as a positive control (*n* = 3, ^*^P < 0.05, ^**^P < 0.01, compared with ADP or thrombin group. ^##^*P* < 0.01, compared with the rest of the groups. Data are expressed as mean ± S.D.).

To further evaluate if DHI could affect platelet activation, we performed an *in vitro* platelet adhesion assay on fibrinogen-coated microplates. As expected, 10 μM ADP or thrombin (0.1 U/mL) caused a maximal adhesion of platelet that was blocked by 2 mM EDTA. As shown in Figure 3A, this ADP-induced platelet adhesion could be dose-dependently inhibited by DHI (1:160–1:20 dilutions) from 100% to 65.55% ± 8.45% – 10.69% ± 7.46%, respectively (*n* = 3, *p* < 0.01). As shown in Figure 3B, thrombin triggered platelet adhesion could be dose-dependently inhibited by DHI (1:640–1:10 dilutions) from 100% to 79.45% ± 4.61% − 7.78% ± 0.13%, respectively (*n* = 3, *p* < 0.01).

### DHI inhibited platelet aggregation *in vitro*

**Figure 4:**
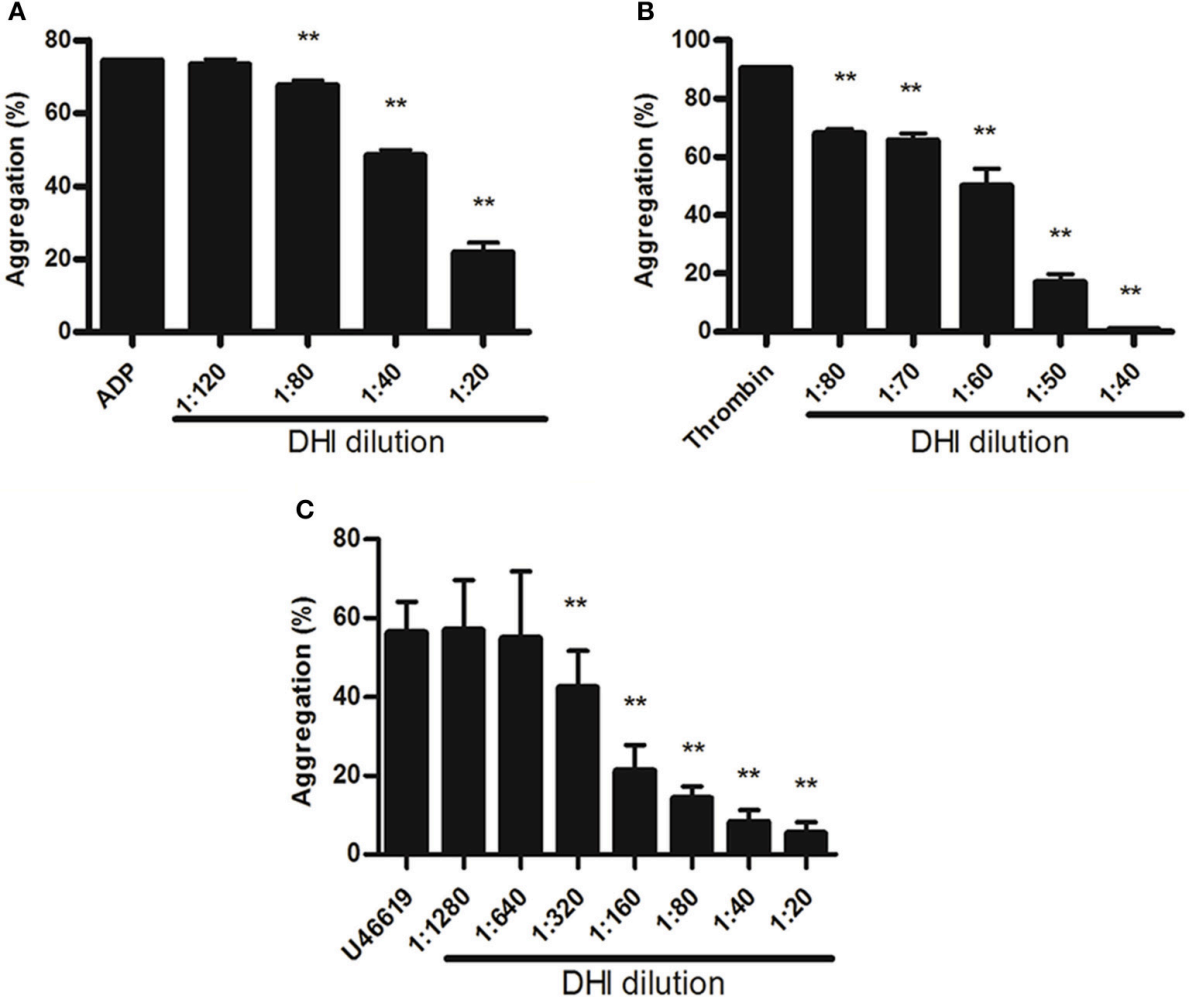
DHI inhibited platelet aggregation. Platelets (1 × 10^8^ platelets/mL) were pre-incubated for 10 min with different concentrations of DHI or vehicle. **(A)** Platelets aggregation was initiated with 25 μM ADP inhibited by series dilutions of DHI (1:120–1:20). **(B)** Platelets aggregation induced by 0.5 U/mL thrombin was inhibited by different dilutions of DHI (1:80 and 1:40). **(C)** Platelets aggregation induced by 11.4 μM U46619 was inhibited by a series dilutions of DHI (1:1,280–1:20, ^**^P < 0.01, All data are expressed as mean ± S.D. *n* = 3).

To further confirm the role of DHI in platelet activation inhibition and to determine if it is mediated by different platelet GPCRs, we performed an *in vitro* platelet aggregation assay using agonists for ADP receptor, thrombin receptor and thromboxane A2 receptor as the platelet activation. As shown in Figure 4A, 25 μM ADP induced a maximal aggregation of platelet which was dose-dependently inhibited by DHI (1:120–1:20 dilutions), from 74.48% ± 0.31% to 73.31% ± 1.44% − 21.87% ± 2.61%, respectively (*n* = 3, *p* < 0.01). Similarly, 0.5 U/mL thrombin triggered platelet aggregation that was dose-dependently inhibited by DHI (1:80–1:10, from 90.35% ± 0.59% to 68.03% ± 1.54% − 4.46% ± 0.24%, respectively. *n* = 3, *p* < 0.01, Figure 4B). Finally, 11.4 μM U46619 induced platelet aggregation was dose-dependently inhibited by DHI (1:1280–1:20) from 64.25% ± 7.81% to 56.89% ± 12.65% − 5.37% ± 2.85%, respectively. *n* = 3, Figure 4C).

### DHI reversed [Ca^2+^]_i_ and inhibition of cAMP production in ADP-activated platelets

**Figure 5:**
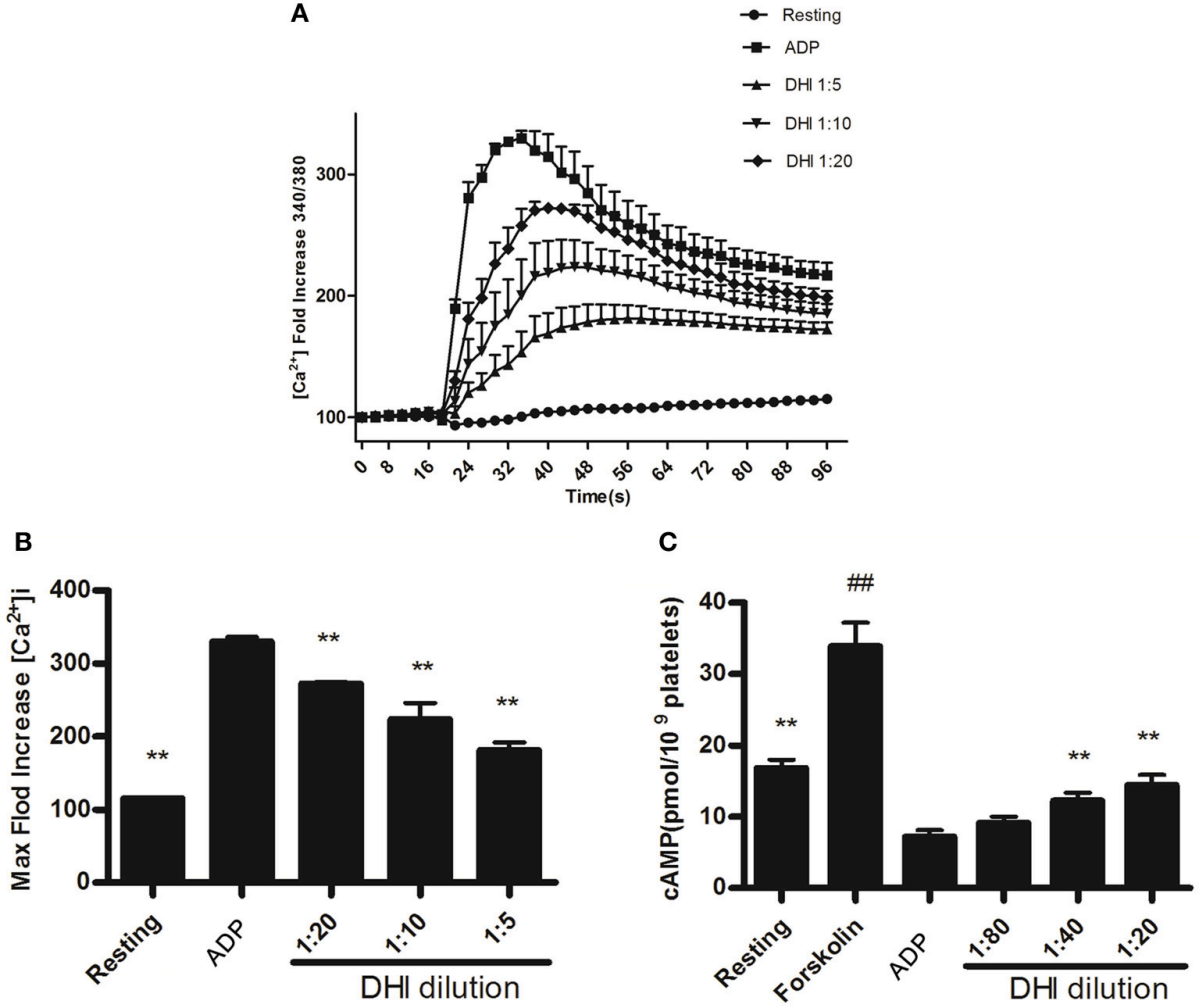
DHI reversed [Ca^2+^]_i_ and inhibition of cAMP production in ADP-activated platelets. **(A)** Time course of different concentrations of DHI on 15 μM ADP induced [Ca^2+^]_i_. **(B)** The maximum intracellular Ca^2+^ produced by 15 μM ADP and inhibited by different concentrations of DHI. **(C)** Reversal of ADP-induced inhibition of cAMP production by DHI. Platelets were incubated with DHI and the control group was incubated with solvent, for 10 min at 37°C, then exposed to ADP (20 μM) for 8 min to cause activation as the control group. Forskolin (10 μM) was used as positive control for cAMP elevation. The cAMP levels were measured by ELISA assay. Previous result has shown that DHI at highest concentration (1:20) had no obvious effect on cAMP in the resting platelet. (All data are expressed as mean ± S.D., ^**^P < 0.01 compared with control, ^##^*P* < 0.01 compared with resting group, *n* = 3).

Since the above platelet adhesion and aggregation results suggested a possible involvement of GPCR in DHI effect on platelet activation, we examined the down-stream signaling pathways of ADP, thrombin, and thromboxane A2 receptors by [Ca^2+^]_i_ and inhibition of cAMP production. As shown in Figures 5A,B, 15 μM ADP led to a maximal [Ca^2+^]_i_[330% relative fluorescence units (RFU)] which was reduced dose-dependently by DHI (1:20–1:5 dilutions) in real time to 275–190%, respectively, *n* = 3). On the other hand, 20 μM ADP inhibited the level of cAMP production from resting (16 ± 1.2 pmol/mL) level to 7 ± 0.9 pmol/mL, which was reversed dose-dependently by DHI (1:80–1:20 dilutions) to 9 ± 0.85 pmol/mL—14 ± 1.43 pmol/mL, respectively, *n* = 3). As a positive control, 10 μM Forskolin stimulated cAMP production at a concentration of (33 pmol/mL, Figure 5C).

### Identification of active components of anti-platelet aggregation activities by screening of a DHI fraction library

**Figure 6:**
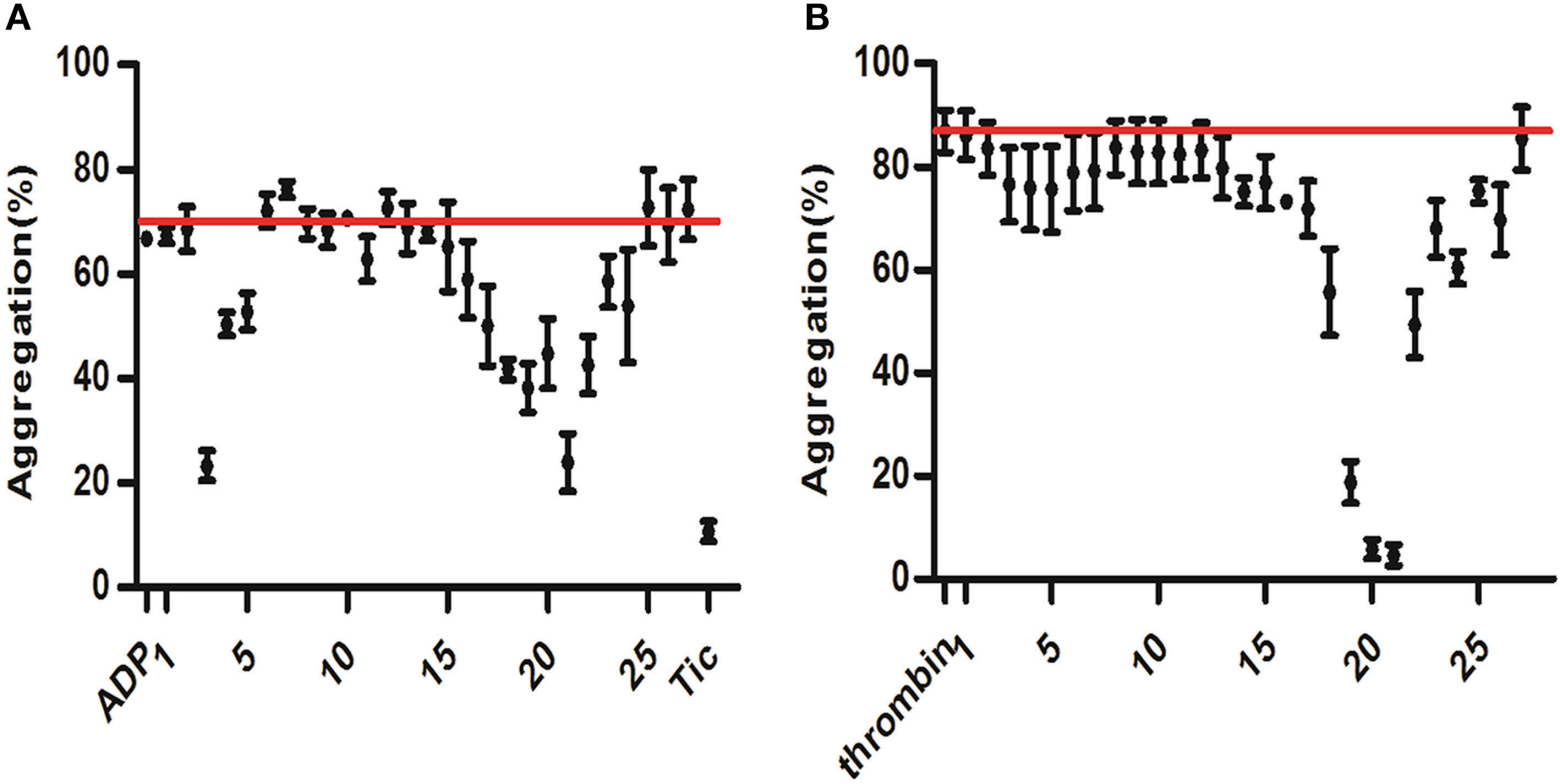
Screening for anti-platelet aggregation activities of DHI fractions. A component library containing 25 fractions of DHI was screened by platelet aggregation assay. **(A)** ADP (25 μM)-induced platelet aggregation of each fraction were measured and plotted. ADP and ticagrelor (Tic) were used as controls indicating maximal activation (66.79%, redline) and inhibition (10.76%), respectively. **(B)** Thrombin (0.5 U/mL) -induced platelet aggregation of each fraction were measured and plotted. Thrombin was used as controls indicating maximal activation (86.86%, redline).

To further evaluate the potential contribution of each fraction in platelet aggregation assay, DHI sample solutions were prepared into 27 fractions via UPLC as previously described (Wang et al., 2016). ADP (25 μM)-induced platelet aggregation of each fraction were measured and plotted. ADP was used as controls indicating maximal activation (66.79% ± 0.03%) and ticagrelor was used as positive control indicating minimal activation (10.76% ± 1.87%). As shown in Figure 6, fractions no. 3 and 21 were the most effective ones in the screening, which inhibited ADP-induced platelet aggregation from 66.79% ± 0.03% to 23.26% ± 2.88% and 23.94% ± 5.54%, respectively. Moreover, thrombin (0.5 U/mL) -induced platelet aggregation of each fraction were measured and plotted. Fractions no. 20 and 21 inhibited thrombin-induced platelet aggregation from 86.86% ± 4.07% to 5.81% ± 1.84% and 4.67% ± 2.04%, respectively. Therefore, the most active fraction no. 21 was selected to reveal the anti-platelet compound identity of DHI.

### Identification of the compounds in fraction no. 21

**Figure 7:**
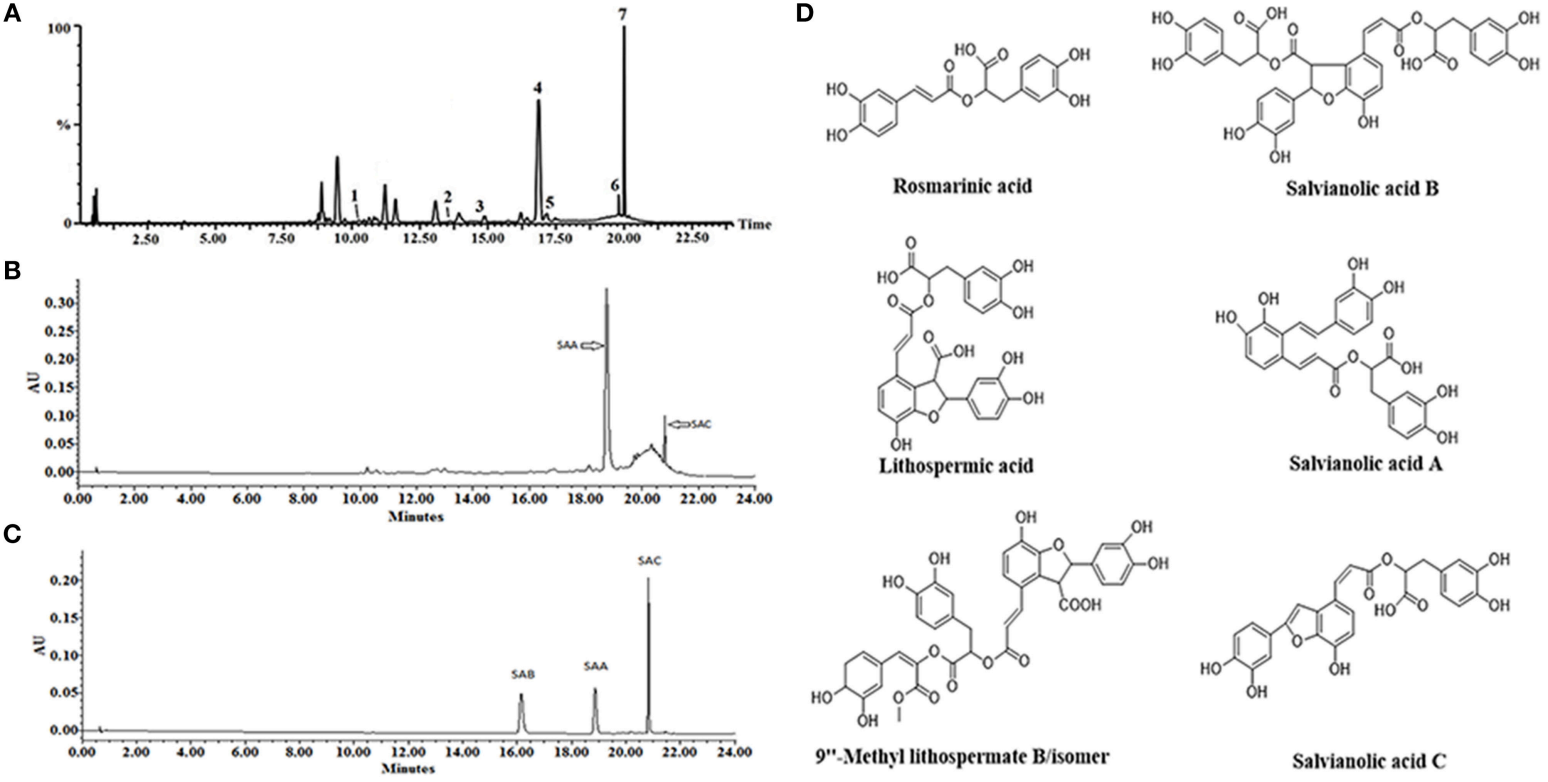
Solid content analysis via LC-MS for identification of the active compounds present in DHI. **(A)** A representative LC-MS chromatogram of fraction no. 21. Polyphenolic compounds, including RA (1), LA (2), SAB (3), SA A (4), 9”- methy lithospermate B/isomer (5,6), and SAC (7) were simultaneously identified. **(B)** A representative UPLC chromatogram of fraction no. 21. SAA (4) and SAC (7) are the main compounds by quantity. **(C)** UPLC chromatogram of SAA, SAB, and SAC reference compounds. **(D)** Chemical structures of the major constituents identified in fraction no. 21.

Based on the result of platelet aggregation screening, the active fraction no. 21 of DHI was further analyzed by UPLC-Q/TOF MS. By comparing the reference compounds and literatures, polyphenolic compounds, including RA, LA, SAB, SAA, 9″- methy lithospermate B/isomer and SAC were identified (Figure 7). As the UPLC chromatogram, SAA (29.17%) and SAC (5.3%) were the main compounds in fraction no. 21. Then, SAA, SAB and SAC were further confirmed with reference compounds.

### Evaluation of interaction between the active constituents

**Figure 8:**
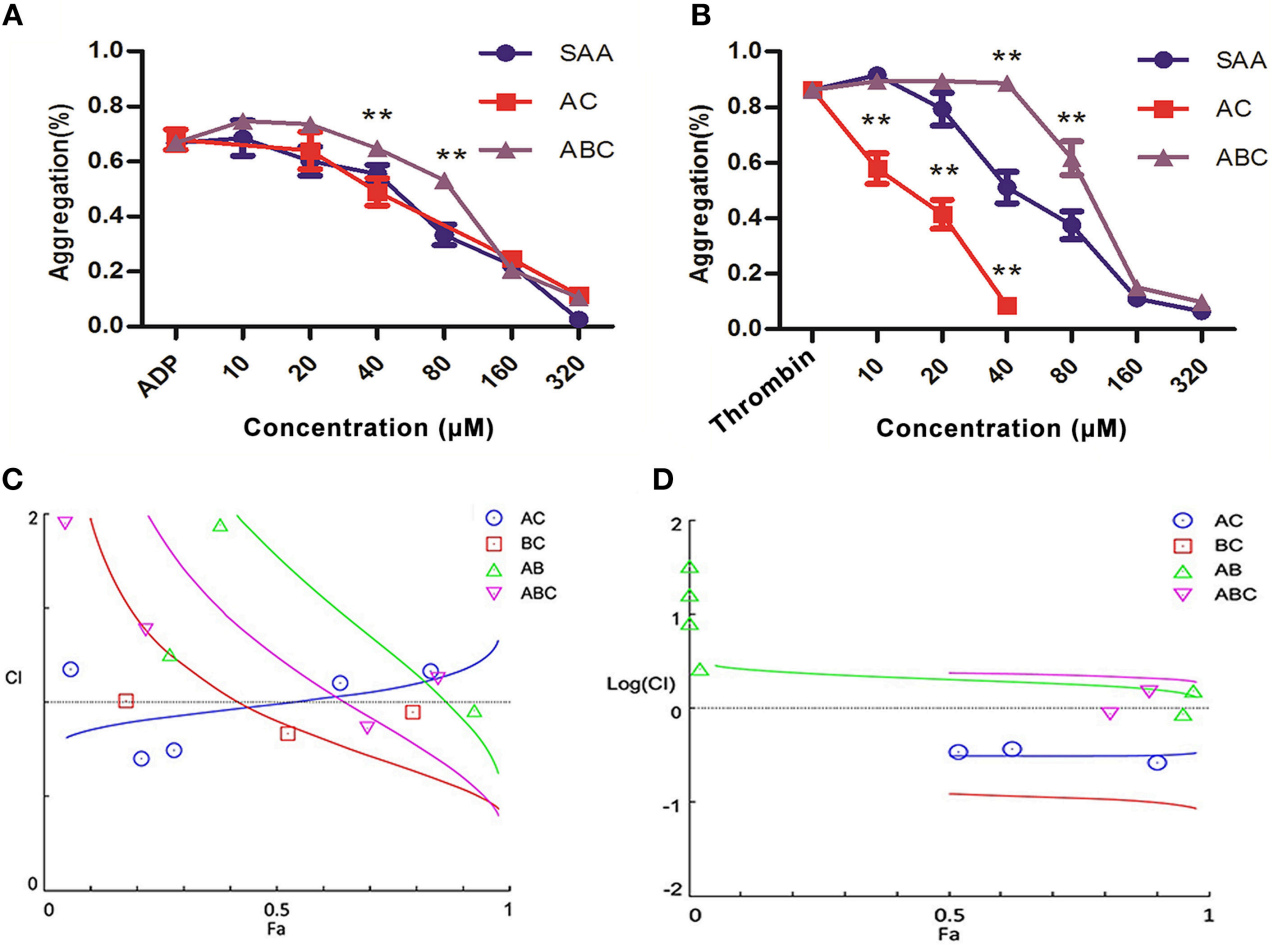
Interactions between active constituents of DHI in platelet aggregation assay. **(A)** Dose-dependent (10–320 μM) inhibition of platelets aggregation induced by 25 μM ADP by SAA, 1:1-ratio mixture of SAA + SAC (AC) or 1:1:1-ratio mixture of SAA + SAB + SAC (ABC). **(B)** SAA (10–320 μM) dose dependently inhibited platelets aggregation induced by 0.5 U/mL thrombin. Also, 1:1-ratio mixture of SAA + SAC (AC, 10–40 μM) or 1:1:1-ratio mixture of SAA + SAB + SAC (ABC, 10–320 μM) dependently inhibited platelets aggregation. **(C)** A combination index (CI)/Fa plot of ADP-induced platelet aggregation. CompuSyn™ software was used to determine the synergy/ additivity/antagonism between SAA, SAB, and SAC. The CI of SAA + SAC or SAA + SAB at Fa_90_ is 1.178 or 0.91, respectively, indicating the interaction between SAA and SAC is antagonism whereas between SAA and SAB is additivity. **(D)** A CI /Fa plot of thrombin-induced platelet aggregation. The CI of SAA + SAC or SAA + SAB at Fa_90_ is 0.325 or 1.60 indicating the interaction between SAA and SAC is synergy and between SAA and SAB is antagonism.

Anti-platelet activity of the main constituents identified above, i.e., SAA, SAB, and SAC (Figure 7D), were evaluated for their interactions individually or in combination, by platelet aggregation assay. The ED_50_ of SAA, SAB, and SAC based on inhibition of ADP-induced platelet aggregation were 69.09 μM (Figure 8A), 214.59 μM and 156.81 μM (data shown in Supplementary Material) in a dose range of 10–320 μM. Then, different compounds mixed in pair or triples with equal concentration were evaluated for their interactions. The ED_50_ of SAA + SAC and SAA + SAB + SAC were 95.44 and 146.45 μM, respectively (Figure 8A). As shown in Figure 8B, the ED_50_ of thrombin-induced platelet aggregation by SAA and SAB were 41.72 μM and 6.19 mM, respectively. SAC had no effect in the same dose range. The ED_50_ of SAA + SAC and SAA + SAB + SAC were 26.07 and 302.1 μM, respectively. The interaction among SAA, SAB, and SAC was further assessed by CI/Fa plot using CompuSyn software. At Fa_90_ SAA and SAC showed a significant synergy for thrombin-induced aggregation with a CI of 0.325 (Figure 8D) but no synergy for ADP-induced aggregation with a CI of 1.178 (Figure 8C). In contrast, SAA and SAB showed a significant antagonism for thrombin-induced aggregation with a CI of 1.60 (Figure 8D) whereas at the same time, an additivity for ADP-induced aggregation with a CI of 0.91 (Figure 8C). Therefore, a combination of SAA and SAC is synergistically the most potent for anti-platelet aggregation while the presence of SAB antagonizes this effect.

## Discussion

In the present study, we demonstrate that DHI effectively dissolved laser-induced arterial thrombus and ameliorated its related symptoms of dry gangrene. We further showed that DHI inhibited GPCR agonists-induced platelet adhesion and aggregation, and reversed G_q_-mediated Ca^2+^ influx and G_i_-mediated inhibition of cAMP production. Furthermore, we identified major active components in DHI for anti-platelet aggregation activities as seven SAs. Finally, we demonstrated that a combination of SAA and SAC synergistically inhibited platelet aggregation while SAB antagonizes this effect.

Although single-targeting anti-platelet agents have been used extensively in clinics, their limitations (bleeding risk and resistance) have started a trend of combination therapy. Drug combinations have been a well-established practice in anti-platelet therapy. For example, dual anti-platelet therapy with aspirin plus clopidogrel has been shown to be more efficacious, and triple anti-platelet therapies with the addition of cilostazol or other agent were also attempted (Michelson, 2010; Spiliopoulos, 2014). *Salvia miltiorrhiza* and *Carthamus tinctorius*, the constituents of DHI, have been reported to either contain active compounds such as CA for direct regulation for the hemostatic and thrombotic system or interact with other anti-thrombotic drugs such as warfarin. As shown in Figure 4, DHI simultaneously inhibited three GPCR-agonists induced platelet aggregation, which revealed that multiple components exist in DHI for inhibiting dual pathways in platelet. These results proved that DHI has a multi-target (thrombin, ADP, and thromboxane) anti-platelet effect, which could serve as a natural combination medicine for thrombosis. Our finding that anti-thrombotic effect of DHI in a rat model was accompanied with alleviation of dry gangrene suggested that DHI could be used clinically for thrombus-related dry gangrene treatment. Of course, all the above potential advantages of DHI for anti-thrombotic prophylaxis and therapy should be fully tested before implemented clinically. Generally, a multi-targeting drug consists of multiple active ingredients (Guan et al., 2013). Although there have been several reports showing that Chinese medicine formulas have the anti-thrombotic activities, our study is the first to show that the anti-platelet activity of a complex Chinese medicine formulas are composed of a group of chemicals by functional screens, in the case of DHI, SAs (Figures 6, 7). However, our data indicates that there may be other components, such as fractions No. 3 and 20, which also contribute to the anti-platelet effect of DHI. Further studies are needed to fully discover these components.

Although our data in Figure 5 showed that DHI had a significant effect on [Ca^2+^]_i_, it did not distinguish if this effect was specific and non-specific. It is well-known that multiple pathways and mechanisms modulate [Ca^2+^]_i_, and several of them have been shown to be affected by DHI ingredients (Spedding and Paoletti, 1992). For example, SAA inhibited L-type calcium current in a dose-dependent manner (Wang et al., 2012). Similarly, SAB has also been shown to inhibiting ADP-evoked [Ca^2+^] increase that act as a P2Y12 antagonist (Liu et al., 2014). Furthermore, CA dose-dependently evoked [Ca^2+^]_i_ increase, which was inhibited by store-operated channel inhibitors such as nifedipine and econazole (Chang et al., 2013). Therefore, at least three different mechanisms were involved in DHI-mediated [Ca^2+^]_i_ change in cells or platelets. The effect of DHI on the [Ca^2+^]_i_ in our experiment may be a combination of multiple substances which need to be further dissected. Our ongoing and future studies will further explore DHI and its individual ingredients on this complicated calcium homeostasis in order to distinguish the GPCR-mediated specific vs. membrane-potential-mediated non-specific effects.

The thrombolytic activity of DHI uncovered in our study could raise a concern for bleeding. However, a recently published post-marketing safety study showed that when a total 30,888 patients in 37 hospitals from 6 provinces were examined, the adverse drug reaction (ADR) of DHI was mainly type A with an incidence rate of 0.35% and without serious concern about bleeding (Li X. L., et al., 2015). According to this study, the main type of ADRs of DHI was type A (including sweating, dizziness, headache, flushing, vasodilation, eye hemorrhage, faintness, chest pain, palpitations, breathlessness, anxious, nausea, flatulence, vomiting, hypotension, hypertension, local numbness, dyspnea, joint disease, and tinnitus) accounting for 57.75%. The severities of most ADRs of DHI were mild and moderate reactions accounting for 25.93 and 66.67%, respectively. Therefore, no serious bleeding incidences by DHI were recorded. DHI is the only prescription CMI that has undergone such vigorous and standard clinical test and provided additional support for the conclusion reached by our study.

Our anti-platelet activity screen and follow-up characterization of different DHI ingredients (mainly SAs) were conducted only *in vitro* and the *in vivo* study used only the whole DHI. However, the reabsorption, elimination and metabolism of a drug may critically influence its activities. The reabsorption, elimination and metabolism of DHI ingredients have been reported by (Li M. et al., 2015). In this study, the specific issue of SAs metabolism was addressed. Specifically, among the 28 catechols detected, the major compounds included TSL, PCA, SAB, RA, SAA, and SAD, and LA. After dosing, TSL, SAD, and LA exhibited considerable exposure in human subjects and rats. However, only TSL had considerable exposure in dogs. PCA and RA circulated in the bloodstream predominantly as metabolites; SAA and SAB exhibited low plasma levels with their human plasma metabolites little or not detected. TSL and SAD were eliminated mainly via renal excretion. Therefore, this study demonstrated that at least PCA and RA are metabolites after DHI administration. These differential metabolisms in different species may lead to different *in vivo* anti-platelet activities which remain to be further explored.

As the most active compound in SAs, SAA has been reported to exert an anti-thrombotic activity by inhibiting PI3K pathway of platelet (Huang et al., 2010). Similar to SAA, SAB has also been shown to inhibit platelets as a P2Y12 antagonist (Liu et al., 2014). Up to now, there is no report on the anti-thrombotic activity of SAC. Our results confirmed the anti-thrombotic activity of SAA but also show that SAC dose-dependently inhibit ADP-induced platelet aggregation. However, we were unable to detect the anti-thrombotic activity of SAB. We believe that the difference was due to platelet source in the 2 studies where Liu's group used human platelet while we used the ones from rat. Interestingly, our results showed that SAB combination with other SAs was even antagonistic in rat washed platelets. This apparent controversy remains to be further explored. For example, ADP receptor or its downstream signaling pathways might have different sensitivity or response in human and rat. Although we and others have shown that SAB is the highest content compound in DHI (Liu H. T. et al., 2013; Liu X. et al., 2013), it showed little anti-thrombotic activity in our experiment. In contrast, SAA has previously been shown to dose-dependently inhibit ADP and thrombin-induced platelet aggregation (Huang et al., 2010), which was confirmed by our current study. It suggested a need to further distinguish the down-stream signaling mechanisms. By [Ca^2+^]_i_ and cAMP assays, we found that SAA selectively regulated down-stream second messenger of ADP-induced platelet activation as indicated by its dose-dependently inhibition of ADP-induced increase of [Ca^2+^]_i_ without regulating the cAMP (data not shown). Furthermore, we showed that the interaction between these SA compounds affected the anti-platelet activity either synergistically or antagonistically (Figure 8). For example, although SAC alone had no effect on thrombin-induced platelet aggregation, its combination with SAA exhibited a stronger effect than the other SA compounds or their combinations in the *in vitro* assay. The inhibition effect of SAA, SAB, or SAC changed when they were mixed together. Further experiments are needed to test the mechanism of the compounds and their mixtures to clarify the interaction between the compounds. It is worth mentioning that the natural abundance and interconversion of SAs may influence the combination effects as our study used a mixture of equal concentration. For instance, it has been reported that SAB could be degraded into SAA and SAA could be degraded into SAC and iso-SAC (Xu et al., 2011). Also, our combination studies were conducted *in vitro* which needs to be further confirmed *in vivo*. Therefore, the more detailed mechanism of the interaction between different SAs remains to be further investigated.

In conclusion, the present study showed that DHI, an effective natural herbal combination, significantly inhibited platelet activation through multiple GPCRs pathways. This effect dissolved artery thrombus and ameliorated dry gangrene. The identification of SAs as a core anti-thrombotic activity of DHI and the discovery that their different combinations could either synergistically or antagonistically provides a better guidance for its safer clinical application and a new direction for further development of natural combination medicine.

## Author contributions

TZ performed all *in vivo* experiments, statistical analysis, co-wrote the paper; LC performed *in vitro* experiments in platelet aggregation, measurements of cAMP and intracellular [Ca^2+^]i; BYZ performed chemical analysis of DHI fractions; ML performed microplate-based platelet adhesion assay; XW performed platelet aggregation screening experiments; XT provided guidance and analyzed the data for platelet aggregation and determination of [Ca^2+^]i; YW supervised the chemistry experiments and contributed the data analysis; GF supervised pharmacological experiments and provided resources; JO assisted in certain experiments and edited the manuscript; BLZ provided guidance to the project; YZ conceived, designed, and supervised the study and co-wrote the paper. All authors read and approved the manuscript.

### Conflict of interest statement

Tianjin University of Traditional Chinese Medicine and Heze Buchang Pharmaceutical Co., Ltd are co-recipients of the following Chinese Ministry of Science and Technology grants: National Major New Drug Discovery (2013ZX09201020) and International Cooperation Project (2013DFA31620). YZ occasionally served as a consultant for the Heze Buchang Pharmaceutical Co., Ltd, China. The other authors declare that the research was conducted in the absence of any commercial or financial relationships that could be construed as a potential conflict of interest.

## Abbreviations

DHI: Danhong injection
GPCRs: G protein-coupled receptors
cAMP: cyclic adenosine- monophosphate
[Ca^2+]^i: intracellular Ca^2+^ concentration
CMI: Chinese medicine injections
PCI: percutaneous coronary intervention
EDTA: ethylenediaminetetraacetic acid
PGE1: prostaglandin E1
UPLC: ultra-performance liquid chromatography
UV: ultraviolet
1H NMR: H-1detection nuclear magnetic resonance
SA: salvianolic acid
TSL: tanshinol
PCA: protocatechuic aldehyde
RA: rosmarinic acid
CA: caffeic acid
LA: lithospermic acid
SAA: salvianolic acid A
SAB: salvianolic acid B
SAC: salvianolic acid C
SAD: salvianolic acid D
PI3K: phosphatidylinositol 3-kinase
SD: Sprague-Dawley rats
ACD: acid-citrate-dextrose buffer
ESI: electrospray ionization mode
LE: leucine-enkephalin
RFU: relative fluorescence units
MS: Mass Spectrum
CI: combination index
Fa: fraction affected
ED50: 50% effective dose.
