# Supplementary material for: Specific Combination of Salvianolic Acids As Core Active Ingredients of Danhong Injection for Treatment of Arterial Thrombosis and Its Derived Dry Gangrene
Source: Front Pharmacol. 2017 Jun 13;8:361. doi: 10.3389/fphar.2017.00361 (PMC5468438; doi:10.3389/fphar.2017.00361)
Supplement: Supplementary file 1 [file DataSheet1.PDF]

Specific Combination of Salvianolic Acids as Core Active Ingredients of Danhong Injection for Treatment of Arterial Thrombosis and Its Derived Dry Gangrene

Running title: Anti-thrombotic mechanisms of Danhong Injection

Tiechan Zhao[1,2], Lianying Chang[1,2], Boyong Zhang[1,2], Ming Lu[1,2], Xiaoyi Wang[1,2], John Owoicho Orgah[1,2], Yuefei Wang[1,2], Xiaoxuan Tian[1,2], Jing Yang[1,2], Guanwei Fan[1,2], Boli Zhang[1,2], Yan Zhu[1,2,3]*

[1]*Tianjin State Key Laboratory of Modern Chinese Medicine, Tianjin University of Traditional Chinese Medicine, Tianjin, China*

[2]*Research and development center of TCM, Tianjin International Joint Academy of Biotechnology & Medicine, 220 Dongting Road, TEDA, Tianjin 300457, China.*

[3]*Molecular Cardiology Research Institute, Tufts Medical Center and Tufts University School of Medicine, 750 Washington Street, Boston, MA 02111, USA*

*Correspondence should be addressed to Yan Zhu, Tianjin State Key Laboratory of Modern Chinese Medicine, Tianjin University of Traditional Chinese Medicine, #88 Yuquan Road, Tianjin, China 300193 Tel.: +86 022 59596168; Fax: +86 022 27386453. E-mail addresses: yanzhu.harvard@icloud.com.

Subject codes: Basic Science Research/ Translational Studies/ Thrombosis

Online Supplementary material

Thrombus Quantification

The experiment begin twelve hours after the last treatment with DHI inject (i.v.). The SD rats were anesthetized with 3% isoflurane in 69% N_2/30% O_2 at continuous inhalation. The previous surgical wound was open and the right external iliac artery was again dissected free of underlining tissues. A small section of artery (4 mm, taking the laser point as the center) which contained laser irradiated point where arterial clot formed was dissected vertically. Then, the artery was flattened on the slide and covered with coverslips. The arteries were imaged using an inverted microscope (Nikon Instech Co. Ltd. Japan). Then, subsequently calculated using the imageJ software (method for calculating the area of the red clot was set in the image).

Diagnostic criteria of the Degree of dry gangrene

The decreased severity order was Gangrene > Tissue ulcer > Cellulitis/Tissue abscess > Tissue (dry and shrunken). The order is based on Wagner's classification system for diabetic foot. The diagnostic criteria of different degree of gangrene were shown below:

Dry gangrene: The affected part is dry, shrunken and dark reddish-black. The line of

separation usually brings auto-amputation. **Tissue ulcer:** appear as open craters, often round, with layers of skin that have eroded. The skin around the ulcer may be red, swollen, and tender. Ulcers develop in stages. In the first stage the skin is red more pronounced, swelling appears, and there may be some blisters and loss of outer skin layers. During the third stage, the skin may become necrotic down through the deep layers of skin, and the fat beneath the skin may become exposed and visible. In the fourth stage, deeper necrosis usually occurs, the fat underneath the skin is completely exposed, and the muscle may also become exposed. In the last two stages the ulcer may cause a deeper loss of fat and necrosis of the muscle. In severe cases it can extend down to bone level, destruction of the bone may begin, and with soft underlying tissue. In the second stage the redness of the skin becomes dark in color there may be sepsis of joints. **Cellulitis:** The area of redness which increases in size over a few days. The borders of the area of redness are generally not sharp and the skin may be swollen. The redness often turns white when pressure is applied. **Tissue abscess:** The abscess area is reddish, warmth and swelling. The swelling may feel fluid filled when pressed. The area of redness often extends beyond the swelling. **Tissue (dry and shrunken):** Skin of the area is shriveled without wet.

Measurement of cAMP using ELISA kit

Note: If the acetylated format of the assay is to be run, all standards (made as introduction of the Kit), samples, and the diluent for the NSB and blank wells must be acetylated as per the instructions in the Reagent Preparation section. Acetylated standards and samples must be used within 30 minutes. 50 µL of Neutralizing reagent was pipetted into each well exception the total activity (TA) and blank wells. 100 µL of the 0.1M HCl was pipetted into the non-specific binding (NSB) and blank (0 pmol/mL standard) wells. 50 µL of 0.1M HCl was added to the NSB wells. 100 µL of Standards No. 1 through No. 5 were Pipetted into the appropriate wells. Then, 100 µL of the samples was also pipetted to the bottom of the appropriate wells. We added 50 µL of the conjugate into each well except the TA and Blank wells. Then, we added 50 µL of the antibody into each well except the blank, TA, and NSB wells. The contents of the wells were emptied and wash by adding 400 µL of wash buffer to every well. The washing step was repeated 2 more times for a total of 3 washes. After the final wash, the wells were emptied by aspirating using a pipette and firmly tapping the plate on a lint free paper towel to remove any remaining wash buffer. 5 µL of the conjugate was again pipetted to the TA wells then, 200 µL of the substrate solution was added into each well. It was incubate for 1 hour at room temperature without shaking. The reaction was stopped by pipetting 50 µL of stop solution into each well. After blanking the plate reader against the substrate blank, the optical density was read at 405 nm.

The cAMP was calculated using the formula below:

$$\mathrm{cAMP}\left(\frac{\mathrm{pmol}}{\mathrm{mL}}\right) = 0.48972/(1 + \left(\frac{\mathrm{OD}}{4.14319}\right)^{0.76228}) - 0.00628$$

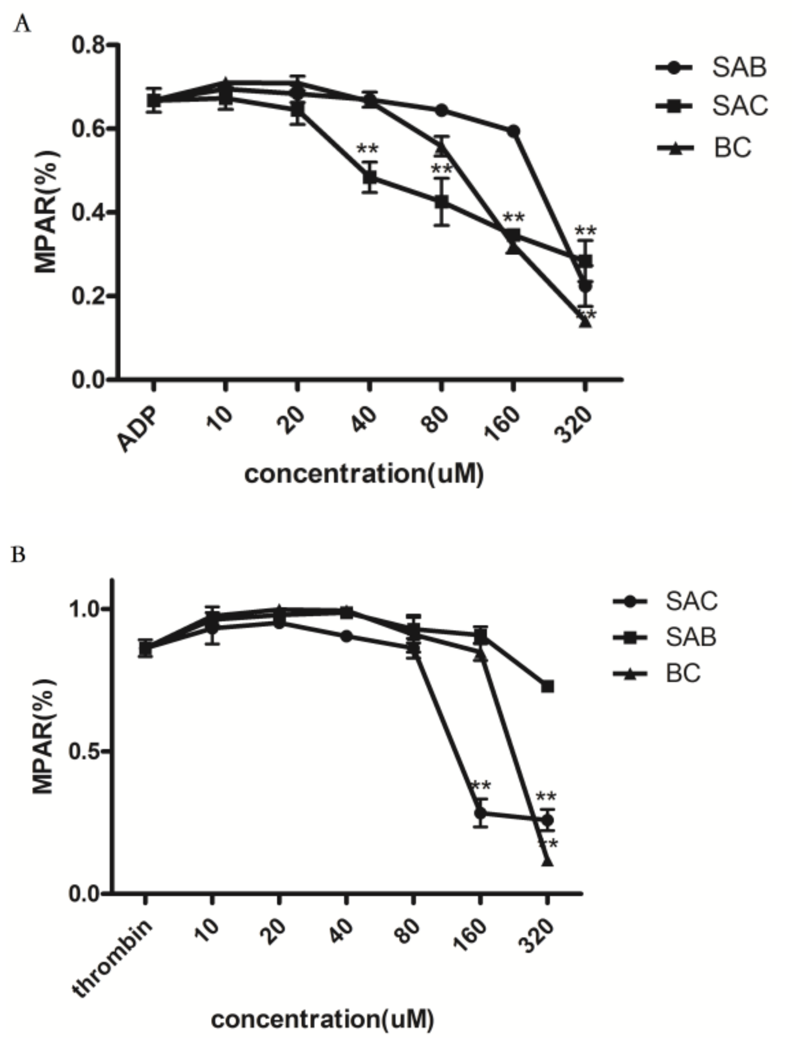

Supplemental Figure S1. (A) Platelets aggregation inhibited by SAB and SAC (10 μM to 320 μM), 1:1-ratio mixture of SAB + SAC (BC). SAB showed no inhibition on ADP-induced platelet aggregation till 320 μM. SAC dose-dependently inhibited ADP-induced platelet aggregation. (B) SAB and SAC (10 μM to 320 μM) inhibited platelets aggregation induced by 0.5 U/mL thrombin. Also, 1:1-ratio mixture of SAB + SAC (BC, 10 μM to 320 μM) inhibited platelets aggregation. SAB showed little effect on thrombin-induced platelet aggregation. SAC did not inhibit thrombin-induced platelet aggregation until 160 μM.

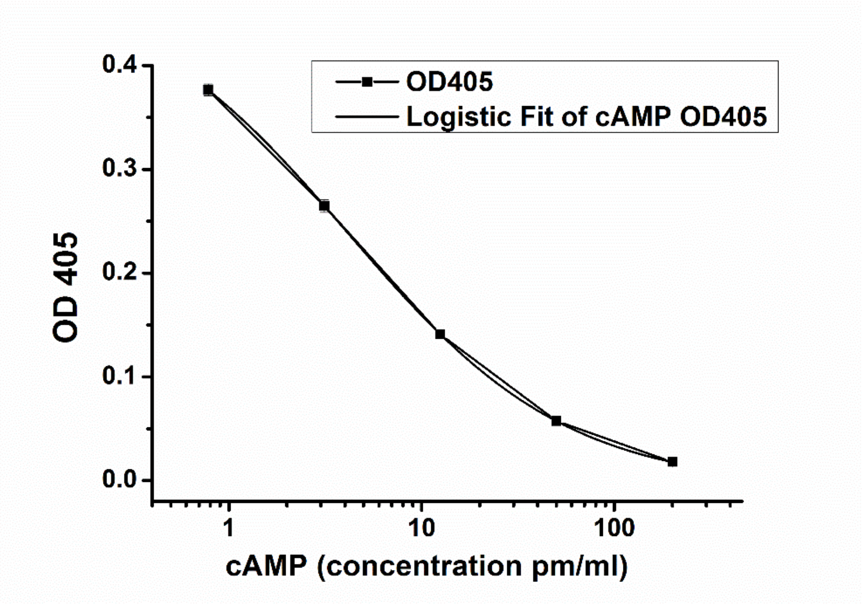

Supplemental Figure S2. Standard curve line of cAMP ELISA assay.
A representation of standard dilution of cAMP sample for ELISA assay provided by the Kit manufacturer.
